# On Spurious Consumption

**Published:** 1872-05

**Authors:** 


### ON SPURIOUS CONSUMPTION.

Dr. D. Francis Condie calls attention, in the *American Journal of the Medical Sciences*, October, 1871, to a disease simulating phthisis, which he calls spurious consumption. He proceeds:

To distinguish spurious from tubercular consumption, the first thing to be taken into account is the lineage of the patient. If on either the paternal or maternal side, or on both, there has prevailed a well-marked strumous diathesis, and a predisposition to the class of diseases to which individuals of that diathesis are liable, we shall have a well-founded reason to infer that the case of consumption, the true character of which is under investigation, is one of tubercular phthisis. Still more so if at the same time the patient is of a decidedly strumous diathesis—as marked by a delicacy of organization, pale countenance, quickly flushed to a pale rosy tint upon the slightest excitement, light colored hair, light blue or grayish eyes, with brilliant whiteness of their adnatæ, very white teeth, great delicacy of skin, showing the subcutaneous veins meandering beneath it, mostly a bulbous condition of the ends of the fingers, with incurvated, scutiform nails; susceptibility to slight degrees of cold, and to the morbific influence of a cold, damp atmosphere, and finally to the subacute character that is assumed by any disease with which they may become attacked.

The pathogonomic symptoms of phthisis pulmonalis occurring in an individual answering to the foregoing description may, with very great certainty, be set down as those of tubercular consumption, more especially when the sounds detected in the chest by auscultation are clearly those appertaining to tubercular disease of the lungs.

It is by no means pretended that the characteristics given above are those alone which distinguish the tubercular diathesis, they are only presented as the most unequivocal. The absence of one or several of them, or their lesser prominency, by no means implies the absence of pulmonary tuberculosis, however much it may embarrass our diagnosis.

An examination of the matter expectorated will often aid us in arriving at a correct diagnosis. In tubercular disease of the lungs, the sputa, in the early stages at least, consist most commonly of a white frothy mucus; later they become more consistent and glairy, and of a darker hue. They are often intermixed with small whitish particles of a cheese like appearance—broken down tubercular matter, and not unfrequently, with distinct masses of a well-defined puriform character.

In what I have denominated *spurious* consumption—consumption without tuberculosis—an individual in the enjoyment of apparently robust health, and without any perceptible predisposition to tubercular disease, will be attacked somewhat suddenly, in most instances after exposure, with acute bronchitis or pneumonia. The disease will, in spite of the best selected and faithfully administered remedial measures, run on in a chronic form for many weeks, or even months, assuming gradually an assemblage of symptoms, which, even with the assistance of the stethoscope, can in many instances scarcely be distinguished, at their height, from those pathognomonic of tubercular consumption. It is only from an attentive study of the entire history of the case that any approach to a certain diagnosis can be made.

It is true that in these spurious cases, the matter expectorated is always more decidedly purulent than it is in the tubercular consumption, while it is entirely free from any fragments of tubercular matter. The peculiar whiteness and bright appearance of the adnatæ of the eyes are seldom present, and are never so prominently marked, as in the tubercular form of consumption. The same statement may be made also in regard to the morbid whiteness of the teeth, the bulbous appearance of the finger ends, and the inverted scutiform shape of the nails. In place of the buoyant, hopeful disposition, and the unclouded intellect, so commonly observed in cases of tubercular phthisis, they who labor under the *spurious* form of consumption are, for the most part, dull, gloomy, dispirited and despondent.

Phisicians who have seen much of consumption under the two forms—the *tubercular* and *spurious*—and have carefully compared the one form with the other, throughout their respective courses will, in the general run of cases, be able to distinguish with sufficient accuracy the one from the other, where others who have neglected this study would entirely fail; and acting upon the diagnosis thus arrived at, they are enabled to effect, in the majority of cases, an entire cure, by entering at once upon an appropriate treatment, rigidly enforced, provided, always, that the patient has come under their care before the destruction of organization in the lungs, and the exhaustion of vitality throughout the system have proceeded to too great an extent to be remedied.—*Half-Yearly Compendium, June*, 1871.

